# An Augmented Reality Device for Remote Supervision of Ultrasound Examinations in International Exercise Science Projects: Usability Study

**DOI:** 10.2196/28767

**Published:** 2021-10-05

**Authors:** Lia Rigamonti, Matteo Secchi, Jimmy B Lawrence, Luca Labianca, Bernd Wolfarth, Harm Peters, Klaus Bonaventura, David Alexander Back

**Affiliations:** 1 University Outpatient Clinic, Sports Medicine & Orthopaedics Department of Sport and Health Sciences, Center of Excellence “Cognitive Sciences”, Faculty of Human Sciences University of Potsdam Potsdam Germany; 2 Italian Association of Hydrotherapists and Newborn Educators (AIIEN) Section of Rome Rome Italy; 3 Department of Health and Physical Education Mercer County Community College West Windsor, NJ United States; 4 Department of Orthopaedics and Traumatology S. Andrea Hospital Sapienza University of Rome Rome Italy; 5 Department of Sports Medicine Charité - Universitätsmedizin Berlin, corporate member of Freie Universität Berlin and Humboldt-Universität zu Berlin Berlin Germany; 6 Department of Sports Medicine Humboldt University Berlin Berlin Germany; 7 Dieter Scheffner Center for Medical Education and Educational Research Charité - Universitätsmedizin Berlin, corporate member of Freie Universität Berlin and Humboldt-Universität zu Berlin Berlin Germany; 8 Department of Cardiology Ernst von Bergmann Hospital Potsdam Germany; 9 Department of Traumatology and Orthopaedics, Septic and Reconstructive Surgery Bundeswehr Hospital Berlin Berlin Germany

**Keywords:** augmented reality, ultrasound, social media, remote, exercise science

## Abstract

**Background:**

Support for long-distance research and clinical collaborations is in high demand and has increased owing to COVID-19–related restrictions on travel and social contact. New digital approaches are required for remote scientific exchange.

**Objective:**

This study aims to analyze the options of using an augmented reality device for remote supervision of exercise science examinations.

**Methods:**

A mobile ultrasound examination of the diameter and intima-media thickness of the femoral and carotid arteries was remotely supervised using a head-mounted augmented reality device. All participants were provided with a link to a YouTube video of the technique in advance. In part 1, 8 international experts from the fields of engineering and sports science were remotely connected to the study setting. Internet connection speed was noted, and a structured interview was conducted. In part 2, 2 remote supervisors evaluated 8 physicians performing an examination on a healthy human subject. The results were recorded, and an evaluation was conducted using a 25-item questionnaire.

**Results:**

In part 1, the remote experts were connected over a mean distance of 1587 km to the examination site. Overall transmission quality was good (mean upload speed: 28.7 Mbps, mean download speed: 97.3 Mbps, mean ping: 21.6 milliseconds). In the interview, participants indicated that the main potential benefits would be to the fields of education, movement analysis, and supervision. Challenges regarding internet connection stability and previous training with the devices used were reported. In part 2, physicians’ examinations showed good interrater correlation (interclass correlation coefficient: 0.84). Participants valued the experienced setting as highly positive.

**Conclusions:**

The study showed the good feasibility of the chosen design and a highly positive attitude of all participants toward this digital approach. Head-mounted augmented reality devices are generally recommended for collaborative research projects with physical examination–based research questions.

## Introduction

### Background

International collaborations play an important role in addressing research questions in almost every scientific discipline [1]. Exercise science is highly interdisciplinary, without regional boundaries, and closely related to different areas of health care. For example, research on vascular changes in athletes [2] can help understand pathologies and global burdens such as cardiovascular diseases [3]. Although sports techniques are often internationally organized and standardized [4], the number of athletes reached for investigations in a single research institution could sometimes be limited depending on the geographic location and the type of sport [5,6]. Multicenter studies are well established in clinical medicine [7,8] and have been used in clinical exercise science research [9]. It would be advantageous to facilitate collaboration among exercise science researchers worldwide for investigations with common research questions, especially with a small population of athletes.

In addition to the impact of the current COVID-19 pandemic with strict travel and contact restrictions and the usual long distances between collaborating institutions, there is a high demand for new digital solutions to bridge distances and contact barriers [1,10,11]. Different digital media or devices may enhance scientific and professional collaborations and thus the health of patients [12,13]. Various approaches have already been described in this context, with early possibilities such as communication or instruction via email or videos [14]. With the rise of the internet and its increasing capability, social media has become highly sophisticated and widespread in the everyday lives of people worldwide [15]. Consequently, this technology is also used for teaching and research purposes depending on individual features, such as direct interpersonal communication, scientific exchange [12], or instructions on medical examinations or surgical techniques [16].

Despite many promising aspects, many of these options lack real-time personalized participation and direct interaction of remote collaboration with partners and examiners in research settings. New digital modes of interaction may be used, such as augmented reality (AR), which can be defined as “technology that integrates digital information into the user’s real-world environment” [17] and is sometimes also referred to as mixed reality [18]. AR devices have already been practically used in the technical settings of business enterprises [19] and for procedural work in space [20]. However, their use in medicine remains experimental. Potential has been described, especially in undergraduate education [21] and in the context of instructing invasive techniques, such as surgery [22]. In some cases, head-mounted AR devices were used in a clinical setting, where magnetic resonance imaging data were made available directly into the vision of surgeons [23]. For example, in sports science, AR has been used to create volleyball court images to measure athletes’ movements in a laboratory setting [24].

### Aim and Hypotheses

This study addressed the feasibility of remote digitally supported exercise science collaborations using two different approaches.

In part 1 of the study, we hypothesized that it is possible to have different experts from various international locations remotely participating in an ultrasound examination setting using an AR device. In addition, their feedback on this method and its potential should be assessed.

In part 2 of the study, we hypothesized that it is possible to have different physicians performing the same ultrasound examination technique while being connected to 2 independent remote supervisors with an AR device.

## Methods

### Study Design

This project consisted of two parts; the same examination technique was used during both parts to obtain ultrasound-based measurements of the intima-media thickness (IMT) and the diameter of the femoral and carotid arteries. All measurements were obtained using the same healthy male human subject. The procedures or measurements were recorded in advance as videos [25]. For better mobility, a portable ultrasound linear array transducer (Lumify; Philips Healthcare) was chosen and linked to a cell phone (HTC Corp) with the appropriate app. The AR device used was a HoloLens 2 (Microsoft Corp) with Dynamics 365 Remote Access software (Microsoft Corp). Connections between participants were enabled by Microsoft Teams software (Microsoft Corp). Participation in the study was voluntary, with no compensation, and anonymity was ensured. All participants provided informed consent. The ethical committee of the University of Potsdam approved this study (EA1/236/19).

### Part 1: Remote Expert Connection and Interviews

In the first approach, different professional experts in engineering and sports science were identified and asked for participation to obtain initial impressions of AR, both from a technical and scientific point of view. None of these participants had previous routine work experience with AR or HoloLens.

**Figure 1:**
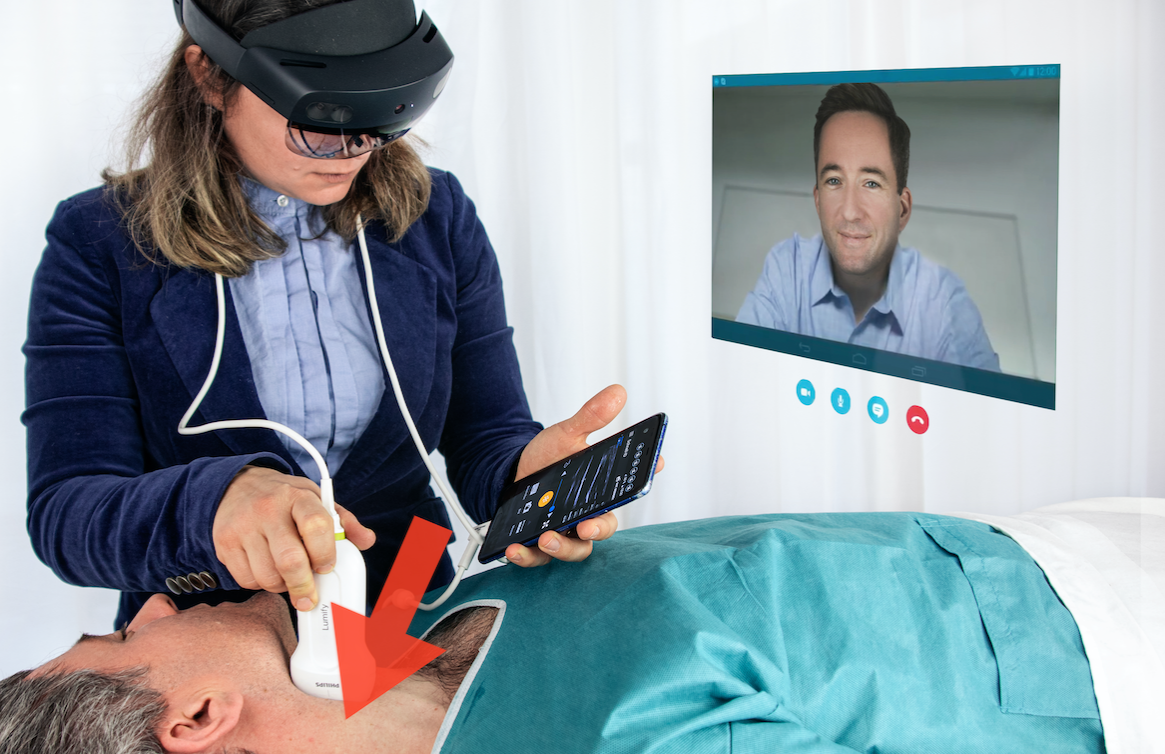
Example of the presented study setting with the investigator wearing the HoloLens 2 (Microsoft Corp) while performing the ultrasound examination with the Lumify transducer (Philips Healthcare).

All participants received an email in advance with a description of the project design and a link to an examination video uploaded on a private YouTube channel, created specifically for this study (YouTube, LCC). After watching the video in advance, the experts then participated separately in the same setting, and the individual experts themselves were located internationally at their chosen location using their personal electronic devices. The physical location where the ultrasound examination was performed was Berlin, Germany. The standardized examination conditions for the human subject are described in the *Examination Conditions* section. Remote live participation was made possible using a Microsoft Teams (Microsoft Corp) digital meeting with the examiner wearing the HoloLens (Figure 1).

**Figure 2:**
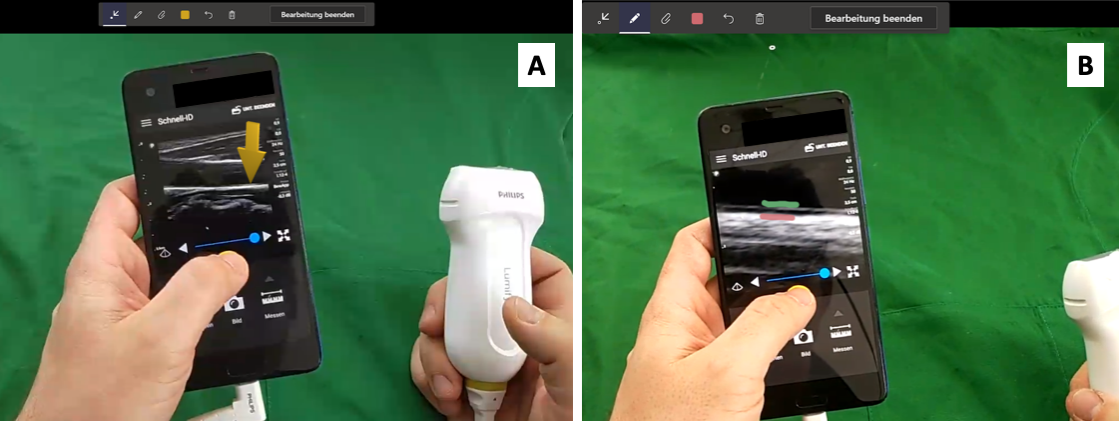
Examples of possible augmented reality indicators viewed while wearing the HoloLens device. (A) Arrow indicating a position to assist where to measure the intima-media-thickness. (B) Green and red lines drawn to indicate where the intima-media-thickness shall be measured.

The experts shared the examiner’s view with ultrasound determination of the IMT and diameter of the femoral and carotid arteries. They also presented the options of drawing arrows or lines into the vision of the HoloLens (Figure 2) and optional blending of pictures or videos into the vision display—features that could be used for visual instruction about precise ultrasound techniques (eg, location of the transducer) or measurements (eg, intrainterventional communication about designated landmarks) in the current setting. The internet download speed (Mbps) and latency or ping (milliseconds) of the observing experts were recorded. Thereafter, they were asked 5 interview questions: (1) written or oral, regarding their perception of social media in general; (2) the remote connection mode in particular; (3) the potential for AR in exercise science and sports medicine; (4) other future digital areas with an impact on this field; and (5) challenges for digitalization in work-related settings.

### Part 2: Examination Setting With Remote Supervision

Before the examination, participating physicians were shown the use of the Lumify transducer along with its mobile app functions and the HoloLens. In addition, they received a link to the unlisted YouTube video, where the examination process and focus data were explained. The participants were asked to wear the HoloLens to measure the diameter and IMT of both the left and right femoral and carotid arteries. In addition, 2 independent remote supervisors were connected with their computer devices by a Microsoft Teams digital meeting to the HoloLens and shared the view of the participating physicians. The performance was rated using the OSAUS (Objective Structured Assessment of Ultrasound Skills) score (maximum 25 points; Multimedia Appendix 1 [26]) and a modified AMIAUD (assessment of mobile imparted arterial ultrasound determination) score (modification: the examiners were not asked to note the results), both recorded by supervisors. This adaptation was made because of the study setting, as the transducer and the mobile phone occupied both examiners’ hands (now maximum 25 points; Multimedia Appendix 2 [25]). Each participant received an evaluation sheet with 25 questions (5-point Likert-scale, multiple-choice, or open-ended questions with text answer options). The topics were as follows:

1. Demographic data (gender, sex);
2. Questions about social media and video-based transmission of knowledge (9 questions; six 5-point Likert-scale, 2 multiple-choice questions, 1 open-ended question);
3. Use of the Lumify ultrasound transducer (5 questions; 4 5-point Likert-scale and 1 open-ended question); and
4. Questions about the practicability and acceptance of the HoloLens device (9 questions; seven 5-point Likert-scale and 2 open-ended questions).

### Examination Conditions

All examinations were performed on the same 39-year-old healthy male human subject lying in a supine position at a mean room temperature of 23 °C. Electrocardiography was conducted during the examination, and blood pressure was assessed before the examination. The subject was asked to consume the same kind of food and avoid caffeine or alcohol 24 hours before each examination to establish standardized conditions.

### Data Analysis

Data were entered in Excel (Microsoft Corporation) and analyzed either descriptively or using SPSS, version 27.0 (IBM Corporation). The interclass correlation coefficient was determined for the interrater correlation of the 2 independent observers of physicians’ examinations. Free text-based answers were checked by 2 of the authors for repetitive sequences. The ratio of the left and right arteries was determined. For vessel measurements, mean values and SDs were calculated; for the scores, median values and quartiles (IQR) were calculated.

The expert interviews were analyzed for relevant topics using MaxQDA 2020 qualitative data analysis software (VERBI GmbH). Predominant themes were defined on the basis of subthemes and their particular topics.

## Results

### Demographic Data

In part 1, the invited sports scientists (2 exercise scientists, 1 physical therapist, and 1 sports physician) were all men and had a mean age of 37 (SD 5.6) years; all engineers (2 automotive engineers, 1 mechanical engineer, and 1 biomedical engineer) were also men and had a mean age of 36 (SD 2.9) years.

In part 2, the 8 participating physicians for the second examination setting (residents from the fields of surgery, orthopedics, anesthesia, and general medicine) were 4 women and 4 men with a mean age of 31 (SD 2.8) years. The remote supervisors were a woman (36 years old) and a man (32 years old).

### Part 1: Remote Expert Connection and Interviews

#### Distance and Internet Connection

The average distance of the remote experts in their different cities in Germany, Italy, England, the Netherlands, Switzerland, and Saudi Arabia to the examination site (Germany) was 1587 (SD 1543) km. The internet connection of all final assessments was stable with a mean upload speed of 28.7 (SD 54.8) Mbps, a download speed of 97.3 (SD 126.3) Mbps, and a ping of 21.6 (SD 11.2) milliseconds.

#### Interview Questions

##### Perception of Social Media

The participants were asked about (1) their perception of the technical aspects of obtaining information via the YouTube video, (2) the connection process to the HoloLens via Microsoft Teams, and (3) the internet connection during the examination. The experts stated that the technical aspects worked well overall, and the processes were easy, straightforward, and easily understood. It was positively reported that access to remote sessions was easily manageable from either a regular computer or mobile phone through a standard telecommunication program such as Microsoft Teams. The YouTube video was rated as highly informative and useful. The connection went smoothly with only one minor connection problem reported when logging into Microsoft Teams and once for a perceived low transmitted voice volume by the HoloLens.

##### Perception of Remote Connection Mode

The general idea of remote digital presence in work- or research-related settings was associated with high approval, also described as an *amazing solution* important for the future; for example, when an expert cannot be physically present at a specific location but is needed. In general, collaboration can be enhanced. Great potential was seen in research teaching and supervision and within business or enterprise. Much of the potential was appreciated because of the current pandemic but also viewed favorably for pre- and postpandemic collaborations across large distances. The use of a relatively common and easy-to-use program may also lower the knowledge barrier required to interact with AR tools. Potential problems of poor internet connection or extended periods to connect were observed. However, compared with lacking alternatives, it is a fast response to urgent situations that require real-time support and allows for worldwide connection. A remote digital presence can be very handy, convenient, and comfortable for individuals.

##### Potential for AR in Exercise Science and Sports Medicine

In the fields of sports science and sports medicine, the experts saw the greatest potential for the use of AR to spread knowledge with teaching and supervise from remote locations. Students and many other recipients (such as athletes) could benefit from this form of knowledge or skill transfer. This could better assist those who benefit from a more *hands-on* or interactive approach.

In sports and training, AR can be used for evaluative or observative analyses, for example, of single movements or patterns, and also as remote medical diagnostics.

A future potential was also described regarding connections with artificial intelligence. AR used concurrently with *smart programs* could, for example, analyze shapes, distances, and track movements (with the possibility of combining this with strength or other external sensors). In addition, deep learning algorithms can provide real-time analyses of an athlete’s performance to coaches, trainers, scientists, and health and sports workers. Thus, beyond observational analysis, physicians could also perform AR-enhanced examinations combined with live data from circulation and tissues.

##### Future Digital Areas With an Impact on Exercise Science and Sports Medicine

Participants were asked which areas and tools will affect sports science and sports medicine the most in the future. Most experts mentioned wearables and smart devices (n=56), followed by mobile apps (n=5), telemedicine (n=5), virtual and AR (n=5), education and training (n=4), artificial intelligence and big data (n=3), and social media (n=2). The participants reported several specific aspects of this technology’s proposed impact: economic, educational (eg, clinical and practical skills training), increased data collection, and increased outreach with the possibility of reaching more people in remote areas.

##### Challenges for Digitalization in Work-Related Settings

**Figure 3:**
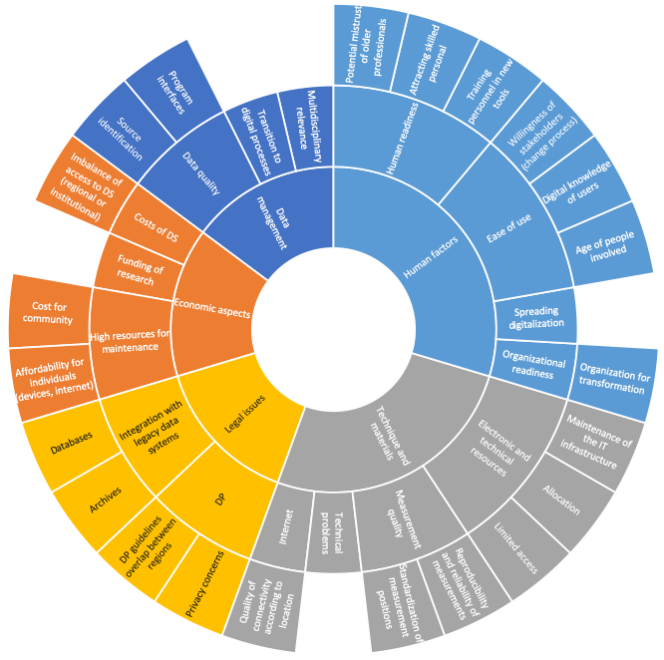
Sunburst diagram showing the areas of challenges for digitalization in work-related settings, as indicated by 8 remote experts. DP: data protection; DS: digital solutions; IT: information technology.

On the basis of the experts’ own experiences, they were asked what the biggest challenges for digitalization in work-related settings will be. The answers provided by the experts are depicted in a sunburst diagram in Figure 3.

### Part 2: Examination Setting With Remote Supervision

#### Gained Examination Data

The mean IMT ratio of the carotid arteries was 1.092 (SD 0.1), and the mean diameter ratio was 1.06 (SD 0.062). The mean IMT ratio of the femoral arteries was 1.032 (SD 0.112), and the mean diameter ratio was 1.001 (SD 0.112). The median of the Objective Structured Assessment of Ultrasound Skills score of the participants was rated at 24.75 (Q1: 23.875; Q3: 25; IQR 1.125) and the mean modified AMIAUD score was 25 (Q1: 24.25; Q3: 25; IQR 0.75), with an interclass correlation coefficient of 0.84 for both raters.

#### Examiners’ Experience Evaluation

During the evaluation of physicians, all stated that they used several social media in their daily lives and had already used videos to acquire professional techniques (mean agreement 4.75/5).

The physician participants considered obtaining video-based knowledge to be practicable in a multitude of capacities. Specifically, the physicians mentioned that it could be used in equipment instruction (n=5), surgical environments (n=4) and emergency rooms (n=2), or further training on examination techniques (n=1).

When asked about the advantages or disadvantages of obtaining knowledge via social media, the participants’ responses were generally positive. They approved that these resources are constantly available, practical, and easily accessible. This enables learning from home or on the road, regardless of location, and repetition of information is easily possible. However, there are some disadvantages. The physician participants mentioned the lack of opportunity for follow-up questions and, in some cases, unclear transparency of the scientific quality.

In response to questions about the advantages and disadvantages of using head-mounted AR devices, the participants stated that it would be advantageous for remote experts to collaborate. This could lead to a gain in knowledge with better technical actions. Furthermore, easy handling and fast data transfer were cited. In contrast, the risk of technical malfunction (especially when *relying* on external help) and the need to learn the handling (eg, head position) were mentioned as disadvantages.

**Figure 4:**
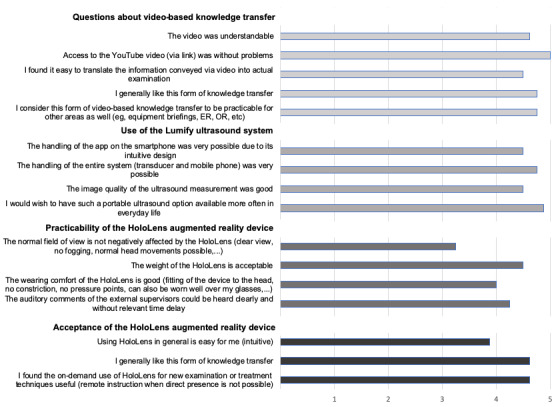
Answers to questions about the video-based transmission of knowledge, the use of the Lumify ultrasound system, and the practicability and acceptance of the augmented reality device HoloLens (n=8 participants; 5-point Likert scale from 1 fully disagree to 5 fully agree). ER: emergency room; OR: operation room.

The other Likert-scale evaluation results concerning the video-based transmission of knowledge, the use of the app- and mobile phone–based ultrasound technique, and the practicability and acceptance of the AR device are shown in Figure 4.

## Discussion

### Principal Findings

Bridging distances between academic institutions and investigators to enhance scientific collaboration has always been highly important for many research projects [1]. Owing to the COVID-19 pandemic, the need to reduce travel and physical contact has reached levels not seen in nearly 100 years [11]. Digital features, such as social media, have already been shown to be sufficient in enhancing scientific exchange and communication [12]. AR technology and its associated devices can positively enhance collaborations across large distances, but the full potential of this technology is not realized, although it is very promising [10].

In this study, remote experts could fulfil the aims and hypotheses and could sufficiently connect to and actively participate in a simulated exercise science examination scenario by collecting the experts’ opinions concerning the techniques used. Furthermore, it is possible that different physicians could perform the given ultrasound examination while being remotely supervised by the AR device in a multicenter international collaboration structure.

Both the experts and the examining physicians showed a high preference for the remote setting using social media and procedural connection modalities via Microsoft Teams. Social media had already been established as a firm tool in the scientific community before the COVID-19 pandemic [12]. Examples of this have been shown when learning about movement techniques in sports and exercise science [27], instructions on investigations [25], and data literacy skills [28]. All these features experienced a boost in the last months of the COVID-19 pandemic when the use of digital communication, digital conferencing, and digital remote working conditions became normal [29].

In addition, the participating physicians shared high ratings regarding the use, practicability, and acceptance of the technologies, although they were not remarkably familiar with the mobile ultrasound device. This was accompanied by a good evaluation of the investigation system and its devices. It seems to have potential as a smart digital transmission feature for ultrasound examinations [30].

AR was rated, in general, as highly positive by both experts and examiners with its previously described potential to establish scientific- and work-related contacts between remote participants in real time and across large distances, providing expert support and exchange, thus enabling international collaborations [10]. In scientific study settings, AR could also benefit from independent and objective supervision by integrating different experts internationally into a rater team, as performed in this study.

The participants saw the potential for AR in sports science and sports medicine first in training and education, which can be supported by the authors’ experiences, for example, in the field of anatomy [21]. Not explicitly mentioned was that the interactivity of AR might make learning more engaging, interesting, and efficient than pure textbooks [31]. Another interesting suggested approach, especially in the field of sports science, could be to link AR with computed tomography or magnetic resonance imaging data to project these data onto moving people, which could support analyses of movement patterns or pathologies and the development of therapies [32]. In general, AR-based human tracking and its analysis seem to be well suited for sports or exercises that depend on form, movement, and technique.

Remote experts and participating physicians stated that good supervision could be possible with AR. Clinical examples show that supervision via AR is possible in surgical settings [10] and that inexperienced users can achieve similar operative results after AR-supervised training compared with other users with on-site training [33]*.*

This study revealed the relevant technical and organizational challenges to be considered. We showed that, with a working internet connection, well-functioning live connections to examinations using AR devices could easily be possible. A decisive point for future projects would be to determine in advance bandwidths or other internet connection conditions that might pose transmission problems [34].

Remotely coordinating the schedules of subject, investigators, and supervisors proved to be difficult. In larger-scale collaborations, multiple meetings should be made available, with significant time frames between each session and optional alternative dates to accommodate all of those involved.

Despite the potential of the described setting, there is a learning curve with this technique in the current form. Users will benefit most after becoming familiar with the devices. The method used in this study involves simultaneously handling the mobile *touchpad* for ultrasound measurements and using a head-mounted AR device. For the latter, the mere weight of the device must be anticipated, among other issues. For example, the way the virtual program appears in the user´s vision is optimal when looking directly forward through the device. The user must anticipate the radius of the camera of the device in relation to the user´s own field of view. Therefore, excessive eye and head movements are discouraged. This aspect has already been described as relevant in the surgical context [35].

The scores used in this study have already been published before [25,26]. Although the quality of the measurements was not the focus, a high correlation was found among the supervisors. Owing to the high rating scores observed, perhaps the ultrasound task was not very challenging for the participating physicians. In addition, this could suggest that there was less need for supervision and auditory comments. Consequently, this might have had an impact on the examiners’ evaluation, although they were asked not only for subjective but also objective answers. It can also be discussed whether AR technology is the only practical choice in this particular setting. Perhaps a head-mounted camera and speaker or microphone could be a sufficient alternative.

However, the chosen study design was intended to serve as an example of the overall practical use of AR devices in an international sports science or medical collaboration setting. Changes in the design according to the scientific requirements and intensified use of AR options will have to be considered for other settings.

Regarding limitations, this study should be viewed as a first step in investigating the feasibility of such technology and techniques because of the small number of participants. In addition, the chosen study design had a stable examination setting with no necessary body movements required by the examiners, thus allowing for good viewing. This could differ in other study designs or real-life scenarios, such as a dynamic setting where numerous and quick body or head movements of an examiner or physician will be necessary (eg, surgical interventions and field research). Furthermore, the long axis of the arteries was used for IMT measurement. However, in future studies, it might be considered that there are fewer influencing factors when performing the measurements on the short axis. Finally, AR options such as showing pictures, videos, and 3D animations (eg, videos in the field of view of an examiner) have not been fully used in this approach.

As there is a lack of larger prospective randomized trials in sports science and medicine that can clearly demonstrate the benefits of AR’s practical applications, the aspects mentioned above and the use of a larger cohort should be considered in future studies.

### Conclusions

This study showed that the described techniques involving social media video distribution in advance, followed by an examination performed with a head-mounted AR device, can be effectively used in a long-distance international collaboration setting. A stable and sufficient internet connection will always be decisive. To optimize international collaboration, especially during the COVID-19 pandemic, this kind of remote support offers advantages such as bridging distances, shortening travel times, providing real-time interaction, and potentially enhancing objectivity in data collection by including remote experts in the study setting. Significant challenges would be the time needed to set up such technical settings, user training, and digital coordination of all participants.

## Appendix

Multimedia Appendix 1 OSAUS (Objective Structured Assessment of Ultrasound Skills) score.

Multimedia Appendix 2 Modified AMIAUD (assessment of mobile imparted arterial ultrasound determination) score.

## Abbreviations

AMIAUD: assessment of mobile imparted arterial ultrasound determination
AR: augmented reality
IMT: intima-media thickness
OSAUS: Objective Structured Assessment of Ultrasound Skills
