# Supplementary figures and images for: An Augmented Reality Device for Remote Supervision of Ultrasound Examinations in International Exercise Science Projects: Usability Study
Source: J Med Internet Res. 2021 Oct 5;23(10):e28767. doi: 10.2196/28767 (PMC8527377; doi:10.2196/28767)

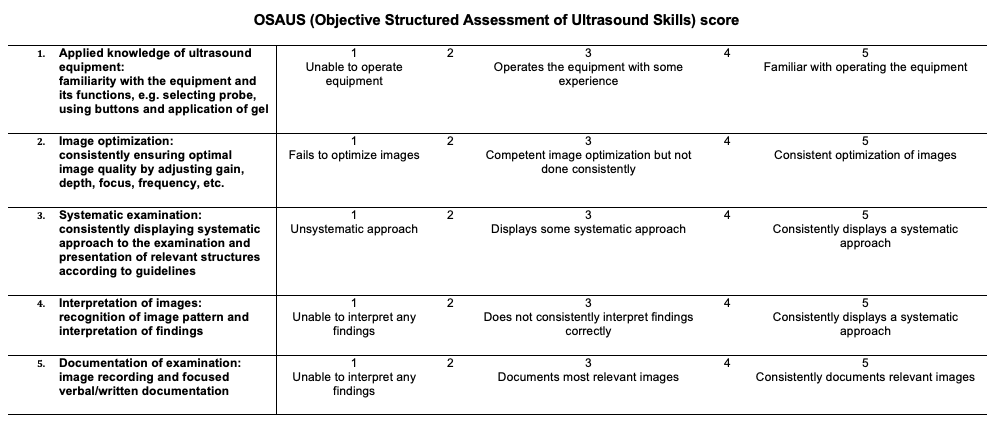

Supplement: Multimedia Appendix 1 [file jmir_v23i10e28767_app1.png]

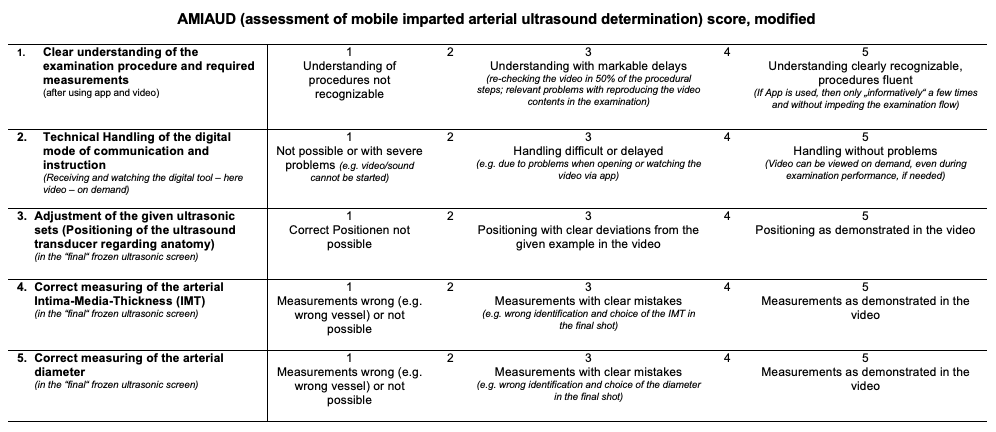

Supplement: Multimedia Appendix 2 [file jmir_v23i10e28767_app2.png]
